# Evaluation of outcomes of a formative objective structured clinical examination for second-year UK medical students

**DOI:** 10.5116/ijme.5572.a534

**Published:** 2015-06-21

**Authors:** Ben Chisnall, Tushar Vince, Sarah Hall, Rachel Tribe

**Affiliations:** 1 GKT School of Medical Education, Faculty of Life Sciences and Medicine, King's College London, UK; 2 Cardiff School of Biosciences, The Sir Martin Evans Building, Museum Avenue, UK

**Keywords:** Formative, summative, OSCE, assessment, clinical skills

## Abstract

**Objectives:**

To explore how formative OSCEs influence student performance and perception when undertaking summative OSCEs.

**Methods:**

We introduced formative OSCEs for second-year medical students at a large London medical school. Examination data from both formative and subsequent summative OSCEs were analysed to determine the effect on summative OSCE performance. We gathered student perceptions using an anonymous online survey tool. The data was investigated using a standard scale of 1 to 5 and qualitative analysis of free text.

**Results:**

Overall, 46.6% and 85.0% of students passed the formative and summative OSCEs respectively. Formative OSCEs did not improve overall pass rates in summative OSCEs. Inclusion of an individual formative station was associated with improved performance in that station in summative OSCEs, with one exception. Formative OSCEs had a positive predictive value of 92.5% for passing the summative OSCE but limited negative predictive value. Students who passed fewer than two out of three formative OSCE stations were significantly more likely to fail the summative OSCE (78.2% vs 89.7%, p <0.001). Students felt formative OSCEs were good exam preparation and suggested logistical changes.

**Conclusion:**

Formative OSCEs were associated with improved performance in subsequent summative OSCEs only for identical stations. They did not improve overall pass rates in summative OSCEs, and did not predict performance well. Students viewed the formative OSCE as a positive and useful activity. However, to maximise its benefit as a tool for learning, students need better communication about the role and purpose of formative OSCEs.

## Introduction

Objective Structured Clinical Examinations (OSCE) are well recognised as a method of clinical assessment. There is good evidence to support the use of OSCEs as a reliable tool to assess clinical competence and determine progress, as well as to improve student performance and confidence, and to enhance enthusiasm for teaching amongst clinical educators.^1^^- ^^3^

OSCEs may be summative or formative, according to their role and purpose in the curriculum. A summative OSCE is an assessment method to formally evaluate clinical skills and knowledge, and constitutes part of the end-of-year or final examinations for the medical degree. Accordingly, students must pass each summative OSCE in order to progress in or complete their medical degree. In contrast, a formative OSCE is primarily a learning tool; it does not contribute to a student's final assessment mark, and “passing” a formative OSCE is not an academic requirement. It may be argued that as a formative assessment is entirely for learning, there can be no “pass” or “fail” decision; however, in order to make most sense of their performance, in our institution, we do provide students with such a judgement. Furthermore, the primary role of formative OSCEs is to familiarise students with the OSCE process and to provide feedback on their performance, thereby enabling improvement before the summative OSCE. The value of such formative assessment in higher education is well documented.^4^

It is important to distinguish between mock OSCEs and formative OSCEs, as they serve different purposes and have different educational outcomes. A mock OSCE replicates the summative OSCE, allowing students to experience the timings, format, layout, length, and station content of a summative OSCE. A formative OSCE, however, may take a different format or length to a summative OSCE, and is primarily designed to enhance learning of clinical and examination skills.^3^^,^^5^^,^^6^ A formative OSCE may be helpful to identify struggling students to enable additional support prior to any summative OSCE. We use formative OSCESs for this purpose in our institution.

There is some evidence that remedial measures after a failed summative OSCE can improve performance in subsequent summative OSCEs and of a positive correlation between performance in early clinical summative OSCEs and subsequent summative OSCEs during medical school.^7^^-^^9^ Mock OSCEs are valued by students and have a positive impact on their confidence and learning experience.^10^ There is little published literature, however, on the introduction and evaluation of formative OSCEs for medical students, especially in the capacity of developing clinical skills and knowledge.

There are important considerations to be taken from the current published literature for the implementation of a formative OSCE. A 2011 study which used formative OSCEs to develop skills of providing and receiving feedback reported that students valued the experience more highly when the educational purpose of the OSCE was made clear to them.^11^ For this to occur, students must understand the rationale behind a formative OSCE, and there is literature to suggest that formative assessment and constructive feedback are most effective when perceived by students to be aligned with the stated outcomes of the course.^12^^-^^14^ These studies emphasise the importance of ensuring that a formative OSCE is designed around curriculum expectations, is aligned with existing institutional examinations, and that these points are communicated to students effectively.

Despite these findings, current literature for what would constitute a successful formative OSCE is limited and there may be uncertainties around the implementation of, format and purpose of, and students' responses to a formative OSCE. Running a formative OSCE is a resource-heavy undertaking and it is important for medical educators to have insights into methods of formative assessment which are effective and useful for student learning. This study aims to address this by examining the introduction of a formative OSCE and evaluating its correlation with results in a subsequent summative OSCE. The findings may be useful for institutions considering the establishment of their own formative OSCEs.

### Background of OSCEs at the study institution

This report describes a case study carried out in a large medical school based in London UK, with approximately 450 students in each year of the Bachelor of Medicine and Bachelor of Surgery (MBBS) medical degree programme.

OSCEs are used as a component of the summative assessment at the study institution from Year 2 onwards, and contribute 16% to the final mark for Year 2. All summative OSCEs also contribute towards awards of merits and distinctions and must be passed in order to progress through the course. Additionally, the OSCEs for Year 3 and Year 4 students contribute to academic rankings for applications to the UK Foundation Programme, which determines placements for the first two years as a junior doctor.  Progression in the summative OSCEs in year 2 is determined by exceeding the pre-set pass mark and passing a specified number of stations (to ensure good all-round competence). Failing the OSCE results in the student undertaking a resit exam of the same format. Failure of the resit will lead to the student failing the year and withdrawing from the course.

Each student is examined by a single examiner in every manned station. Given the size of the student cohort, we run two identical circuits simultaneously for the summative OSCE and between three (2013 cohort) and four (2014 cohort) identical circuits simultaneously for the formative exam. However, in order to maintain consistency all examiners (and simulated patients) involved in a particular station are given protected time immediately before the exam to standardise the simulated patients’ performances and for examiners to agree what the student must demonstrate in order to achieve the score, thereby standardising the marking process.

The content of each OSCE is determined by the curriculum but may be regarded as the standard skills of demonstrating practical skills and procedures, conducting a physical examination, taking a history or explaining management to a real or simulated patient. The number of active stations in summative OSCEs we use is 16 in the second and third years, 17 in the fourth year and 18 in the final year. Stations vary in duration from 5 minutes in the second year to 7.5 minutes in the final years and a further 1-2 minutes between stations for examiners to write feedback. In addition to a checklist, a "global mark" is awarded to students by the examiner based on their fluency and mastery. Simulated patients also provide a global score on the level of communication skills – this is effectively taken as patient feedback. Both groups receive training in the regard.

A modified Angoff process is used for standard setting, with the standard set at "safe minimum competence" for the relevant year of study. External examiners are involved in standard setting. Examiners are briefed on each day of the OSCE on the standard expected. Thereafter the examiners standardise their marking. In addition to the examiner guidance provided for each station, this process acts as its own moderation. As such, the pass mark is pre-set before the exam and no adjustments are made to marks after the exam.

### Demographics at the study institution

The gender breakdown is 54.0% female and 46.0% male, with 91.9% of students from the UK or EU and 8.1% from non-EU countries.There are approximately 300-350 students who enter directly from secondary school education, around 30 on the dedicated four-year graduate entry programme, and around 50 students on an extended degree as part of a widening access to medicine programme. The programme runs for six years rather than the usual five, allowing the first stage to be studied at a slower pace and with greater support for the first three years. These students follow the same medical curriculum as all other medical students and undergo the same rigorous assessment. The programme is only eligible to students within a certain geographical location of the institution and attending non-selective state schools.

### Pass marks and feedback

For both formative and summative OSCEs, a pass mark for each station is set in advance of the examination. The standardised overall pass mark for both formative and summative OSCEs is 50 out of 100. As described above, in the summative OSCE, the standardised mark also reflects the number of stations passed – this requirement to pass a certain proportion of stations is well-recognised and attempts to ensure students have good all-round knowledge and skills. If a candidate does not pass a sufficient number of stations, the standardised mark is capped at 49. This was not applied in the formative OSCE as it comprised too few stations to make this a meaningful component of the results.

For both formative and summative OSCEs, students receive feedback on their performance on the day the exam results are released (one to two weeks after taking the exam). This feedback is in three different formats: domain feedback based on station type; ‘hot feedback’ written by the examiner in the short interval between stations which provides indicators of performance and suggestions for improvement; and cohort feedback for the whole year, which focuses on performance trends across all students.

### Purpose of the study

In 2013, a formative OSCE was introduced for medical students in the second year of their degree programme to enhance clinical education and assessment, and to address a number of student concerns. This was a response to previous cohort feedback that students felt unprepared for their summative OSCE at the end of their second year. In particular, students reported unfamiliarity with the format of the exam. The formative OSCE was designed to allow students to experience OSCE conditions, as well as to practise this form of assessment and to receive constructive feedback to inform their further learning.

This study aimed to evaluate the introduction of a formative OSCE with comparison to student performance in a subsequent summative OSCE. This evaluation was conducted through analysis of student cohort performance data and interpretation of anonymous responses to an on-line questionnaire. Use of both qualitative and quantitative methods was regarded as important to assess both the actual effect on students' pass rates and their perceptions of the educational value of the experience; the questionnaire also enabled students to suggest modifications and improvements to the formative OSCE in future years. 

## Methods

Two successive cohorts (2012-3 and 2013-4) of second year medical students at a UK medical school were encouraged to participate in a non-mandatory formative OSCE two months before their summative OSCE. The students received a briefing on the formative OSCE two weeks beforehand, which outlined the purpose of the exam and gave generic guidance in how to approach the stations. The formative OSCE was run at a single site, with multiple simultaneous circuits over five days. It comprised three stations of five minutes each, with an additional five minutes observing another student perform a station. Examiners were given 2.5 minutes between stations to write feedback. All students in the first formative OSCE (2013) undertook the same three stations: living anatomy, urine dipstick testing, and administration of a subcutaneous injection. Similarly, all students taking the 2014 formative OSCE undertook the same three stations: living anatomy, urine dipstick testing, and blood pressure measurement (Table 1). The formative OSCE was carried out under examination conditions, with clinical examiners and simulated patients as used in the summative OSCE, and with standardised mark sheets and feedback. Performance data was obtained from student records.

 We reviewed the success data in the two formative OSCEs with performances in their respective summative exams. The summative exams were run at the same geographical site over a four to five day period; each day would have six circuits in total (three sessions of two simultaneous circuits). In addition, we compared the data to summative OSCE performance from 2012, which preceded the implementation of the formative OSCEs. As a control measure, we also compared the performances in a range of summative OSCE stations that did not appear in the formative OSCE.

### Differences in stations between formative and summative OSCEs

The list of stations used in the formative OSCEs is given in Table 1. Two of the three formative stations (the urine dipstick testing and subcutaneous injection stations) followed a similar format in the formative and summative OSCEs, so the formative experience closely resembled the summative examination. One station in each formative OSCE (the living anatomy station in 2013 and the blood pressure measurement station in 2014) differed from its format in the summative OSCE.

**Table 1:** Formative OSCE stations

Station	2013	2014
Station 1	Urine testing	Urine testing
Station 2	Subcutaneous injection	Anatomy
Station 3	Anatomy	BP measurement
Observation	Observe anatomy station	Patient having BP measured

The living anatomy station in both formative OSCEs required students to discuss an anatomical diagram to the examiner and answer questions, with an emphasis on correlating anatomy to clinical presentations. The anatomy station in the 2013 formative OSCE differed from the 2014 formative station in that it involved a student observing the fluency of another student and to provide feedback. The formative station also differed in format to the summative OSCE in that the anatomy stations in the summative OSCE were surface anatomy, where students demonstrated clinically-relevant anatomical landmarks on a simulated patient (for example demonstrating the landmark for inserting an intercostal chest drain), and unmanned anatomy stations, where students answered multiple choice questions (MCQs) based on a diagram, bony specimen or anatomical model. The latter station format was closest to the formative station.

For the blood pressure station in the 2014 formative OSCE, the student demonstrating the procedure would then act as the simulated patient for the next student, and would give feedback accordingly. In the summative OSCE, we use a simulated patient for all students.

### Questionnaire

Student opinions were collected from an online survey tool asking five questions about their experience of the formative OSCE and how this affected their performance in the summative OSCE.  The questions originally formed part of the student evaluation of the course. This survey allowed anonymous responses to four statements on a 5-point Likert scale, and there was an additional question allowing free text responses.  Both cohorts of students were e-mailed a link to this online survey after their summative OSCE in May, but before they received their results in June. The questions asked in the survey are included in Table 2.

### Statistical analysis

OSCE performance data were analysed using Microsoft Excel 2007 and differences between data sets were assessed using Chi-squared testing of variance; significant differences were accepted when p<0.05. This methodology was chosen to allow comparisons of the proportions of students passing or failing individual stations or the examination as a whole. Although OSCE marks are standardised within a single year of students, this standardisation depends on the range of marks within the cohort and means comparisons of standardised marks between different years is less helpful than pass/fail percentages. Quantitative and qualitative analysis of the questionnaire material allowed triangulation of the study findings.

This study used existing institutional protocols for anonymised academic data collection and anonymous student feedback. All data used in this study were previously collected as either examination data or student evaluation data. Furthermore, data collection and analysis conformed to British Educational Research Association guidelines.^15^ Therefore, in accordance with local policy, this study did not require local ethics approval.

**Table 2:** Questions used in survey

Question
The formative OSCE helped me to prepare for the summative OSCE
I felt confident going into the summative OSCE
I was less anxious going into the summative OSCE because of my experience of the formative OSCE
I think I have done better in the summative OSCE compared to the formative OSCE

## Results

### Student performance in formative and summative OSCE examinations

Across the two years of this study, 774 students sat both the formative and summative OSCE: 361 in 2013 and 413 in 2014. Of these, 361 students passed the formative OSCE (46.6%) and 658 students passed the summative OSCE (85.0%). All performance data are summarised in Table 3. For the formative OSCE, the mean standardised mark out of 100 was 51.2 (SD = 11.3), and for the summative OSCEs the mean standardised mark was 59.7 (SD = 8.8). The median standardised marks were 49 and 61 for the formative and summative respectively.

The Cronbach's alpha for the 2013 summative exam overall was 0.66, varying from 0.56 to 0.79 on individual days; the Cronbach's alpha for the 2014 summative was 0.59, varying from 0.51 to 0.66 on individual days. We were unable to identify meaningful reliability data for the formative OSCEs, given the small number of stations.

Of the 361 students who passed the formative OSCE, 334 (92.5%) subsequently passed the summative OSCE, and 27 (7.5%) failed the summative OSCE. Of the 413 students who failed the formative OSCE, 324 (78.5%) subsequently passed the summative OSCE, and 89 (21.5%) failed the summative OSCE. This gave a positive predictive value of 92.5% for passing the formative OSCE as a predictor of passing the summative OSCE, and a negative predictive value of 21.5% for failing the formative OSCE as a predictor of failing the summative OSCE. However, the data demonstrated that he higher the numbers of stations passed in the formative OSCE, the more likely students were to pass the summative OSCE (Table 4). For students who passed 0-1 stations in the formative OSCE, 78.2% went on to pass the summative OSCE, compared to 89.7% of students who passed 2-3 stations in the formative OSCE (p < 0.001).

We reviewed the pass rate in summative 2012 OSCE (before the implementation of the formative OSCE) and compared this with the overall pass rates for the 2013 and 2014 summative OSCEs. This indicated that there was no statistically significant difference between the proportions of students passing in each year. The pass rate in 2014 was 87.2%, compared to 84.4% in 2012 (p=0.30) and the pass rate in 2013 was 82.6% compared to 84.4% in 2012 (p=0.50) (Table 5). For comparison, we reviewed the performance in summative OSCEs of a group of stations that do not appear in the formative OSCEs and again there were no significant differences.

**Table 3:** Comparison of OSCE performance in formative and summative examinations

Variable	Total(n = 774)	2013 (n = 361)	2014 (n = 413)
Formative OSCE			
Passed exam, n (%)	361 (46.6)	180 (49.9)	181 (43.8)
Mean mark (SD)	51.2 (11.3)	51.7 (11.6)	50.8 (11.0)
Median mark	49.0	49.0	49.0
Summative OSCE			
Passed exam, n (%)	658 (85.0)	298 (82.6)	360 (87.2)
Mean mark (SD)	59.7 (8.8)	59.6 (8.0)	59.8 (9.5)
Median mark	61	60	61
Comparative performance			
Passed formative, passed summative, n (%)	334 (43.2)	163 (45.2)	171 (41.4)
Passed formative, failed summative, n (%)	27 (3.5)	17 (4.7)	10 (2.4)
Failed formative, failed summative, n (%)	89 (11.5)	46 (12.7)	43 (10.4)
Failed formative, passed summative, n (%)	324 (41.9)	135 (37.4)	189 (45.8)

**Table 4:** Percentage of students passing the summative OSCE compared to number of stations passed in the formative OSCE

Formative Stations passed	% Passing summative total	% passing summative 2013	% passing summative 2014
0	76.8	76.0	77.4
1	79.0	76.4	81.0
2	87.5	84.3	90.6
3	94.5	91.3	97.4

### Effects of formative experience in specific OSCE stations

Students in 2014 had the blood pressure measurement station as part of their formative OSCE, whereas students in 2013 did not, and there was no formative OSCE for students in 2012 (Table 5). A significantly greater proportion of students in the 2014 summative OSCE passed the blood pressure measurement station than students in either 2013 (69.3% vs 52.0%, p <0.001) or 2012 (69.3% vs 56.9%, p <0.001).

**Table 5:** Percentage of students passing each station type in the summative OSCE by year (2012 to 2014)

Summative OSCE Station	Pass rate for summativeOSCE stations (%)		
	2012	2013	2014
BP Measurement	56.9	52.0	69.3*
Subcutaneous Injection	76.5	87.1*	87.7
Urine Testing	96.8	96.8*	74.2*
Anatomy MCQ	75.6	74.7	75.4
Skeletal Anatomy	84.9	78.4	86.2
Surface Anatomy	69.8	48.8	64.7
Explaining	68.5	45.6	57.7
Exploring	57.1	62.8	66.7
Focused history taking	62.7	79.7	79.0
History Taking	87.6	81.8	69.3
Sensory Awareness	69.6	83.4	86.2
Overall Pass Rate	84.4	82.6	87.2

*Values shown in bold are those stations also covered in the formative OSCE in that year (no formative OSCE in 2012)

For the subcutaneous injection station, a significantly greater proportion of students passed in the summative OSCEs in 2013 and 2014 than in 2012 (2013: 87.1%vs 76.5%, p < 0.001; 2014: 87.7% vs 76.5%, p <0.001). Although students in 2013 had the subcutaneous injection station as part of their formative OSCE, whereas students in 2014 did not, there was no significant difference between the proportions of students passing this station in the summative OSCEs in 2013 and 2014 (87.7% vs 87.1%, p = 0.80) (Table 5).

For the urine dipstick testing station, the pass rate in the summative OSCE was the same in 2012 and in 2013 (96.8%), but dropped in 2014 to 74.2% (p <0.001), despite both 2013 and 2014 cohorts experiencing the urine dipstick station in formative and summative OSCEs.

For anatomy stations in the summative OSCE, a significantly lower proportion of students passed the skeletal anatomy station in 2013 than in either 2012 (78.4% vs 84.9%, p = 0.03) or in 2014 (78.4% vs 86.2%, p = 0.007). There was no significant difference in the proportion of students passing the skeletal anatomy station between 2014 and 2012 (86.2% vs 84.9%, p = 0.63). This was similar for surface anatomy: a significantly greater proportion of students passed in 2014 than in 2013 (64.7% vs 48.8%, p <0.001) and a significantly greater proportion passed in 2012 than in 2013 (69.8% vs 48.8%, p <0.001). There was no significant difference in the proportion of students passing the surface anatomy station between 2014 and 2012 (64.7% vs 69.8%, p = 0.12). For the unmanned anatomy MCQ station, there was no significant difference in the proportions of students passing across the three years 2012, 2013 and 2014. (Table 5)

### Student perceptions of the formative OSCE

A total of 308 out of 786 students responded to the online survey, giving a response rate of 39.1%; 195 out of 361 in 2013 (54.0%) and 113 out of 425 in 2014 (26.6%). The results are summarised in Table 6.  The majority of respondents found the formative OSCE a valuable experience in helping to prepare for the summative OSCE, as well as reducing anxiety about the examination (Table 6).

The final question of the survey provided an opportunity for respondents to provide free text comments about the formative OSCE. Of the 308 survey respondents, 250 (81.2%) provided comments and a summary of these are given in Table 7. Overall, the most common response from students was that the formative OSCE helped with preparation for the summative exam, by allowing familiarisation with exam conditions and logistics (42.8% of responses), or was "useful" in some way (28.8% of responses). One hundred students (40.0%) said that they would have liked more stations in the formative OSCE, and a minority of students gave negative comments, which related to the relevance of the formative OSCE itself (18.4%) or the feedback received (8.8%). In general, a greater proportion of the students in 2013 perceived the formative OSCE as a positive learning experience, whereas more negative comments were received from the 2014 cohort.

**Table 6:** Student responses to online survey about experience of formative OSCE*

Question	Agree Total(%)	Disagree Total(%)	Agree 2013(%)	Disagree 2013(%)	Agree 2014(%)	Disagree 2014(%)
The formative OSCE helped me to prepare for the summative OSCE	70.6	14.9	76.0	9.9	60.7	22.3
I felt confident going into the summative OSCE	28.9	39.3	31.2	32.8	25.7	51.3
I was less anxious going into the summative OSCE because of my experience of the formative OSCE	56.8	24.4	64.0	18.2	46.0	35.4
I think I have done better in the summative OSCE compared to the formative OSCE	52.3	19.3	50.0	18.8	57.1	20.5

*Agree = percentage of students choosing "Strongly Agree" or "Agree"; Disagree = percentage of students choosing "Strongly Disagree" or "Disagree"

**Table 7:** Main themes in free text responses by students

Main themes	Total% (n)n=249	2013% (n)n=158	2014% (n)n=91
Good exam preparation	42.8 (107)	44.9 (71)	39.6 (36)
More stations needed	40.0 (100)	36.7 (58)	46.2 (42)
Useful experience	28.8 (72)	31.6 (50)	24.2 (22)
Poor/unhelpful feedback on performance was received	18.4 (46)	13.9 (22)	26.4 (24)
Observation station was unhelpful	14.8 (37)	13.9 (22)	16.5 (15)
Format was unrealistic and/or unrepresentative	8.8 (22)	3.8 (6)	17.6 (16)
Students felt badly informed about format	5.2 (13)	2.5 (4)	9.9 (9)
Good feedback on performance received	2.4(6)	3.2 (5)	1.1 (1)

## Discussion

### Comparison of formative and summative OSCE results

Although the introduction of the formative OSCE did not result in a significant change in the overall pass rate of the summative OSCE, the improved performance in individual stations suggests that the formative examination experience may have had a beneficial educational effect for the students.

The formative exams differed in content over the two years. This was partly done to minimise the effect of students knowing the content before the exam and partly in response to changes in timetabled teaching. Whilst this is a potential limitation of the study, it had the advantage of eliminating prior knowledge as a factor for success in the summative OSCEs.

The 2014 cohort of students achieved higher pass rates at the blood pressure measurement station in the summative OSCE compared to the 2013 or 2012 cohorts, after they had experienced this station as an observation and feedback station in their formative OSCE; this may support an improvement based on constructive feedback and the chance to practice the station under exam conditions. Similarly, the 2013 cohort improved on their predecessors' performances in 2012 for the subcutaneous injection station, suggesting that the presence of this station in the formative OSCE had an educational benefit. It is not clear why this improvement was sustained for the 2014 cohort, although communication between students in a higher year is fostered at the study institution through a cross-year buddying system which may have had some impact on dissemination of advice on OSCE preparation. There was a less clear pattern of improvement for the anatomy stations, but this may be due to the format differences between anatomy stations in the formative and summative OSCE, in particular the greater focus on basic anatomical knowledge over clinical implications of anatomy in the summative stations. There was no improvement in pass rates for the urine dipstick station in the summative OSCE between 2012 and 2013, despite the 2013 cohort having practised the station in their formative OSCE, and a decline in performance from 2013 to 2014, with the 2014 cohort also having experienced the station in their formative OSCE. Although the format of the station was kept consistent between 2013 and 2014, the different pass rates may be a result of the content of the station changing in the summative OSCE between these two years from diabetes and glycosuria to hypertension and proteinuria, despite both topics being taught in detail.

From this study, students showed a significant improvement in performance in particular stations in the summative OSCE when they had a chance to practice these in the same format and with the same knowledge requirements in the formative OSCE, compared to students who had not had a formative OSCE. However, the fact that there were improved pass rates for stations in the summative OSCE that did not appear in the formative OSCE suggests that more research is needed to determine the value of the formative experience in influencing summative OSCE performance.

The formative OSCE has a good positive predictive value but a poor negative predictive value, meaning that students who pass the formative OSCE are likely to pass the summative OSCE, but the majority of students who fail the formative OSCE are also likely to pass the summative OSCE. In 2014, out of all the students who passed the summative OSCE, more than half had previously failed the formative OSCE, and when aggregated across the two years, the proportions were roughly equal. The numbers of students who failed the formative OSCE, therefore, is not a particularly useful method of predicting who will fail the summative OSCE. This would be expected if the experience of the formative OSCE is educationally beneficial, and supports the value of the formative exercise.

A better predictor of performance in the summative OSCE is the number of stations passed in the formative OSCE. Specifically, students who passed only one station in the formative OSCE were more likely to fail the summative exam; this suggests that it may be helpful to target more specific interventions or support to this group.

The small number of stations in the formative OSCE was a limitation in our study; three active stations and one observation station for each student was the maximum possible with the time and resources available. In addition, it served the underlying purpose of giving the students an opportunity to experience the OSCE as a logistical exercise. Previous research has shown that reliability in the use of OSCEs to assess clinical competence only approaches an acceptable standard when the exam is a minimum of 2 hours or has at least 10 stations.^1^^,^^16^ Three stations do not provide sufficient information to accurately measure a student's clinical competence, and is one reason why it is difficult to predict performance in the summative OSCE from the formative result. However, reasons for the improvement in performance between formative and summative OSCEs include increased motivation from a poor mark in the formative OSCE, improved exam techniques from the chance to practise, and development of clinical skills during the interval between formative and summative exams. There was no evidence available for the minimum number of stations that would be required for a formative OSCE to have a significant educational effect and further research is necessary to determine the optimal format, length and composition for a formative OSCE designed to offer a learning experience relating to the examination itself, rather than to predict clinical competence directly.

### Students' perceptions of the formative OSCE

In general, the formative OSCE was viewed as a positive and useful activity, with the majority of students applying their learning experience to their preparation for the summative OSCE.

The perceived value of the formative OSCE was supported by the students' desire for more stations in the formative OSCE. Some students compared the formative OSCE with mock OSCEs run by student societies, which were felt to be more true to the summative OSCE than the formative OSCE (citing the observation stations in the formative OSCE as an example of this disconnect). This highlights the importance of clearly communicating the role of a formative OSCE and how it contrasts to a mock examination. Only one student in our study mentioned the positive learning opportunities of the formative stations. This may indicate that the students have an inadequate understanding of the purpose of the formative OSCE, which could be improved with better advanced communication to the students. Our institution has taken large steps in this regard.

With the exception of the observation stations, the formative OSCE was kept as similar in format as possible to the summative OSCE, as the study institution emphasises the importance of constructive alignment across formative and summative assessment. This alignment allowed students to experience the feel and format of the summative OSCE beforehand, and provide familiarity with the OSCE layout prior to the summative exam. Yet this constructive alignment may have contributed to some students' lack of clarity regarding the purpose of the formative OSCE. Although our formative OSCE was intended as an educational experience, its constructive alignment perhaps gave the impression that it was a mock OSCE, and in this respect our formative OSCE was more of a hybrid between a formative and a mock. The fact that so many students were expecting a full mock, as well as their negative comments about the observation station, again emphasises that better communication may be needed in future.

From the survey responses, the 2014 cohort expressed fewer positive comments about the formative OSCE than the 2013 cohort, and was more critical of its length, organisation and content. As the formative OSCE was run in the same manner, and two out of the three stations were identical across the two years, it may be that the second cohort in 2014 had higher expectations of the formative OSCE than their predecessors, for whom it was a new feature of their course and no preconceptions existed. An additional difference between the cohorts was that a proportion of the 2014 survey responses arrived after the publication of the summative OSCE results, whereas all of the 2013 responses were received before summative OSCE results were released. The low response rate to the survey, however, means that subgroup analysis of the questionnaire responses is difficult to interpret accurately.

## Conclusion

Our study shows that although the introduction of a formative OSCE did not have a significant impact upon pass rates for the summative OSCE; it may have had a beneficial effect on students' learning of certain clinical skills as measured by OSCE performance. Students clearly appreciate the opportunity to practise their clinical and examination skills under the 'low-risk' conditions of a formative exam. Whether students pass or fail a three-station formative OSCE is not sufficient to predict which students will go on to fail a subsequent 16-station summative OSCE, but assessing the number of stations each student passed may be a better predictor of summative performance. Quantitative analysis of the effect of a larger formative OSCE on summative pass rates would be a useful contribution to the current literature.

Although the majority of students found the formative OSCE a positive experience, there were some misconceptions about its purpose, indicating that these students predominantly viewed its role as preparatory for the summative OSCE rather than as educationally beneficial in its own right. This may be helped by better communication to students and clearer definition of the purpose of the formative OSCE, as well as its relationship to the summative OSCE.

### Acknowledgements

BC was funded by a King's College London Undergraduate Research Fellowship for his work on this paper.

### Conflict of Interest

The authors declare that they have no conflict of interest.
